# Translational Analysis of Effects of Prenatal Cocaine Exposure on Human Infant Cries and Rat Pup Ultrasonic Vocalizations

**DOI:** 10.1371/journal.pone.0110349

**Published:** 2014-10-22

**Authors:** Philip Sanford Zeskind, Matthew S. McMurray, Elizabeth T. Cox Lippard, Karen M. Grewen, Kristin A. Garber, Josephine M. Johns

**Affiliations:** 1 Department of Pediatrics, Carolina Healthcare System's Levine Children's Hospital, Charlotte, North Carolina, United States of America; 2 Department of Pediatrics, University of North Carolina, Chapel Hill, North Carolina, United States of America; 3 Department of Psychology, University of Illinois at Chicago, Chicago, Illinois, United States of America; 4 Department of Psychiatry, Yale University, New Haven, Connecticut, United States of America; 5 Department of Psychiatry, University of North Carolina, Chapel Hill, North Carolina, United States of America; Texas Christian University, United States of America

## Abstract

Spectral and temporal features of human infant crying may detect neurobehavioral effects of prenatal cocaine exposure (PCE). Finding comparable measures of rodent ultrasonic vocalizations (USVs) would promote translational analyses by controlling the effects of correlated variables that confound human studies. To this end, two studies examined the sensitivity of similar acoustic structures in human infant and rat pup vocalizations to effects of PCE. In Study 1, cry sounds of 107 one month-old infants were spectrum analyzed to create a novel set of measures and to detect the presence of hyperphonation - a qualitative shift to an atypically high fundamental frequency (basic pitch) associated with neurobehavioral insult. Infants with PCE were compared to infants with prenatal polydrug-exposure (PPE) without cocaine and with infants in a standard comparison (SC) group with no prenatal drug exposure. In Study 2, USVs of 118 five day-old rat pups with either PCE, prenatal saline exposure or no prenatal exposures were spectrum analyzed to detect the presence of frequency shifts – acoustic features that have a frequency waveform similar to that of hyperphonation. Results of study 1 showed PCE had two sets of sex-dependent effects on human infants: PCE males had higher pitched cries with more dysphonation (turbulence); PCE females had longer pauses between fewer cry sounds that were of lower amplitude than comparison groups. PCE and PPE infants had more cries with hyperphonation than SC infants. In study 2, PCE pups had a greater percentage of USVs with shift in the acoustic structure than pups in the two control groups. As such, the novel measures of human infant crying and rat pup USVs were sensitive to effects of PCE. These studies provide the first known translational analysis of similar acoustic structures of vocalizations in two species to detect adverse effects of prenatal drug exposure.

## Introduction

Maternal cocaine-use during pregnancy continues to be a significant public health concern [1]–[3] with subtle effects [4] having far-ranging implications for the health and development of exposed children [5]–[10]. Prenatal cocaine exposure (PCE) has adverse neurochemical, vasoconstrictive and dysregulatory effects [11] that may be evident in early neurobehavioral deficits [12]–[18] and problematic mother-infant interactions that may further contribute to poor developmental outcomes [19]–[22]. The independent role of PCE in the development of these neurobehavioral deficits, however, is less than clear due to the many confounding conditions, such as poverty and prenatal polydrug exposure (PPE), that are often a part of the larger context of maternal cocaine-use during pregnancy [23], [24]. Translational analyses of the effects of PCE that provide experimental control over such confounding conditions would be facilitated by finding sensitive measures of neurobehavioral function that are comparable across human and other species. The purpose of this paper is to examine the utility of a novel set of spectral characteristics of human infant cry sounds and measures of rat pup ultrasonic vocalizations (USVs) in a translational analysis of the effects of PCE on early neurobehavioral development.

Spectrum analysis of the acoustic and temporal features of human infant cry sounds has long been used to detect adverse effects on neurobehavioral integrity of a wide range of prenatal and perinatal conditions, extending from cases of obvious brain damage and genetic disorders [25], [26] to prenatal exposures to opiates [27], marijuana [28], tobacco [29], and alcohol [30]. Cries of infants with these and other nonoptimal pre- and perinatal conditions typically include a higher fundamental frequency (F_0_ or basic pitch), greater amounts of dysphonation (sonic turbulence), shorter initial expiratory sounds and variations in several other measures of the power spectrum. A hallmark of these distinctive cry sounds is the frequent presence of hyperphonation - a sudden, qualitative shift to a high-pitched acoustic structure with an F_0_ over 1000 Hz – that occurs more often in response to physically intense eliciting conditions [31]. Analyses of up to the first three cry expirations following a painful stimulus have shown these acoustic features to differentiate cry sounds of infants with PCE [12], [32], [33], but effects on specific measures have been inconsistent across studies and differences between PCE and comparison infants may no longer be evident when statistical analyses are adjusted for polydrug covariates [12]. Little is known, however, about the value of an analysis of cry sounds beyond the initial three cry expirations. For the present study, we developed a novel set of spectral and temporal measures designed to capture the presence of hyperphonation and dynamic characteristics in an extended period of infant crying.

Spectrum analyses of rodent USVs have also shown that variations in such measures as amplitude, F_0_, number, and repetition rate may reflect adverse effects of prenatal and postnatal drug exposures on early neurobehavioral development [34], [35]. Genotypic-dependent effects of PCE on the number and beginning pitch of USVs of infant mice have been described [36], but no effects of PCE have been found in rodent pup USVs using measures directly comparable to those used in studies of human infant cry sounds [37]. In contrast to measuring variations in specific acoustic features, another approach has been to examine categories of USV waveforms based on the pioneering taxonomy described by Brudzynski and colleagues [38]. Neonatal exposure to alcohol, for example, has been shown to selectively reduce the presence of specific waveform categories in mouse USVs [39], but no effects of cocaine have been found using this methodology. Interestingly, a rarely noted USV waveform, not included in the above taxonomy, has an acoustic structure similar to that of the hyperphonation found in human infant cry sounds. Described in the USVs of rat pups and other rodent species as steps or shifts [40], [41], this waveform is characterized by a qualitative shift in F_0_, in the absence of a vertical line in the spectrogram within the same USV. Anecdotal reports suggest that, like hyperphonation in human infant cries, steps or shifts occur more often in physically intense eliciting conditions [42] and may also reflect effects of drug exposure on neurobehavioral regulation [43]. Parallels between the acoustic structures of hyperphonation in human infant crying and shift in rat pup USVs may provide the basis for a translational analysis of vocalizations in the study of effects of PCE on neurobehavioral integrity. In the present study we systematically explored the presence of frequency shifts in the vocalizations of rat pups under intense thermal conditions and whether this acoustic waveform was differentially represented in the USVs of rat pups with PCE.

## Methods

### Study 1: Human infant crying

#### Subjects

Pregnant women and mothers of infants younger than 4 weeks of age were recruited via community advertisements, drug rehabilitation centers and social services as part of a larger study of the neurobehavioral and social effects of prenatal cocaine exposure. All human procedures were approved by the University of North Carolina's Office of Human Research Ethics Biomedical Institutional Review Board (IRB). An IRB-approved written Parental Permission for a Minor Child to Participate in a Research Study (Biomedical) was obtained for infant participation, and was signed by each infant's biological mother prior to testing. A signed written consent form approved by the University of North Carolina's Biomedical Institutional Review Board was also obtained from all mothers in order to collect maternal and prenatal characteristics prior to testing. Infants of these mothers (n = 131) participated in a cry analysis paradigm at approximately one month of age (days: M = 27.3, SD = 13.3) and were assigned to one of three groups for comparison. PCE infants (n = 31) had mothers who were currently involved in drug-treatment services, had self-report of cocaine-use during pregnancy on a Time Line Follow Back (TLFB) questionnaire, and/or had prenatal urine samples positive for cocaine use at the level of 0.300 ng/ml for cocaine and metabolites. PCE infants were also exposed prenatally to maternal use of antidepressants, alcohol, cigarettes, marijuana, and opiates during pregnancy. A standard comparison group (SC) was comprised of infants (n = 50) whose mothers did not use antidepressants and had a negative urine toxicology for cocaine, marijuana, methamphetamine, or opiates at laboratory visit, did not endorse drug-use on a phone screen, and/or did not test positive for drug use. A novel, second comparison group was comprised of infants (n = 38) with prenatal polydrug-exposure (PPE) if their mothers used tobacco, alcohol, marijuana, opiates and/or antidepressants in varying degrees, but not cocaine. Chi square analyses showed that PCE and PPE infants did not reliably differ in the distributions of prenatal exposure to antidepressants, alcohol, cigarettes, marijuana or opiates (all p's>.25).

Table 1 shows maternal demographic and infant growth characteristics of this sample. Compared to mothers in both the PCE and PPE groups, mothers in the SC group were disproportionately Caucasian, as compared to African-American [*X* ^2^(2) = 8.07, p<.018], married [*X* ^2^(6)  = 33.5, p<.001], and had a higher level of attained education [*X* ^2^(10)  = 51.97, p<.001]. No group differences were found in methods of delivery (Vaginal vs. C-section) [*X* ^2^(2)  = 3.85, p<.15]. One-way analyses of variance showed that the three groups of infants differed in birth weight [F(2,128)  = 10.91, p<.001] and gestational age [F(2,128)  = 6.63, p<.002]. LSD post-hoc tests showed that PCE infants had a lower birth weight and gestational age than infants in the two control groups. PPE and SC infant groups did not differ on these measures (all p's>.20). Infant birthweight was significantly correlated with gestational age (p<.001), maternal education (p<.001) and maternal ethnicity (p<.05). Infant groups also did not differ in post-conception age at the time cries were recorded [F(2,129) = .198, p<.82], or in the distribution of males and females (PCE: males  = 20, females  = 19; PPE: males  = 20, females  = 22; SC: males  = 30, females  = 20) [*X* ^2^(2) = 1.51, p<.48].

**Table 1:** Human Subject Demographics.

Demographic		Comparison	Cocaine	Polydrug
Number of subjects		38	31	38
Maternal Ethnicity	Caucasian	34 (68%)	25 (64.1%)	17 (40.5%)
	African-American	12 (24%)	10 (25.6%)	20 (47.6%)
	Hispanic	1 (2%)	2 (5.1%)	20 (7.1%)
	Asian	2 (4%)	0 (0%)	1 (2.4%)
	Other	1 (2%)	2 (5.1%)	1 (2.4%)
Maternal Marital Status	Divorced	1 (2.3%)	6 (17.2%)	4 (11.8%)
	Married	24 (55.8%)	1 (2.8%)	5 (14.7%)
	Never Married	17 (39.6%)	24 (68.6%)	21 (61.7%)
	Separated	1 (2.3%)	4 (11.4%)	4 (11.8%)
Maternal Education	≤ High School	10 (22.7%)	16 (44.4%)	13 (39.5%)
	Some College/Trade School	6 (13.7%)	20 (55.6%)	14 (42.4%)
	College Graduate	14 (31.8%)	0 (0%)	4 (12.1%)
	Post Graduate Education	14 (31.8%)	0 (0%)	2 (6.0%)
Delivery Method	Vaginal	40 (80%)	33 (87%)	29 (69%)
	C-Section	10 (20%)	5 (13%)	13 (31%)
Infant Birth Weight (g)		3506.8±52.4	3081.5±67.4	3388.9±78.2
Gestational Age (weeks)		39.9±0.2	38.9±0.3	39.7±0.2
Age at Assessment (days)		24.5±1.0	30.2±2.1	25.6±1.1

Note: Values are raw numbers, with percentages in parentheses, and Mean ± SEM where appropriate.

#### Cry recording and analysis

Crying was elicited by placing infants on a cold metal scale maintained at 20°C and recorded using an Olympus DM-20 digital recorder (44.1 kHz sampling rate) held 20 centimeters vertically and mid-sternum horizontally from the infant's mouth. The initial 30 s of the crying bout was downloaded to a Multi-Speech Lab (Kay/Pentax) computer software program and down-sampled to 22,050 Hz for greater resolution (±21 Hz) of frequencies up to 11 kHz. Digital spectrograms were produced for each crying bout and examined by a research assistant blinded to infant group and sex. Twenty-four infants did not cry (PCE n = 8, SC n = 12, PPE n = 4), thus resulting in a total of 107 infants whose cries were analyzed (PCE n = 31: males  = 15, females  = 16; PPE n = 38: males  = 18, females  = 20; SC n = 38: males  = 26, females  = 12).

**Figure 1:**
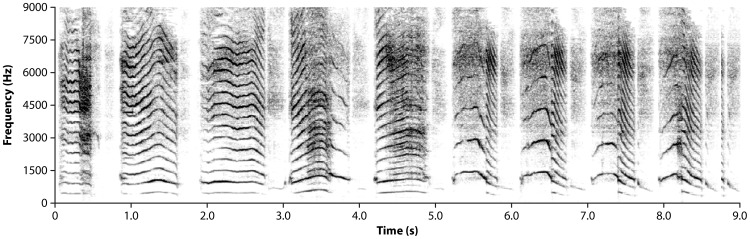
Spectrographic Display of Human Infant Crying. This figure shows the standard phonated harmonic structure of crying evident in the first 3 expiratory cry sounds, followed by expiratory cry sounds with hyperphonation, evident at the end of the fourth cry expiration and in cry expirations that occurred after 5 sec. A greater vertical distance between harmonic frequencies denoted by horizontal lines characterizes this acoustic structure. This figure is an example of finding the highest Peak F_0_ in the crying bout beyond the initial three cry expirations often used in previous studies of cry analysis.

As described in previous work, measures of crying were derived from the temporal and spectral characteristics of each expiratory cry sound produced during the entire 30 s recording period [44]. Peak F_0_ (in Hz), and its amplitude (F_0_ Amp) in decibels (dB), were determined from the power spectrum of a Fast-Fourier Transform (FFT) conducted on the 25 ms sample at which F_0_ reached its highest point in each expiratory cry sound. The multiple assessments of Peak F_0_ and F_0_ Amp were summed and averaged for an overall mean value. F_0_SD was calculated as the standard deviation of the multiple assessments of Peak F_0_. Initial F_0_ was the highest Peak F_0_ found in any one of the first three cry expirations. Durations of temporal measures (seconds) were determined via cursor placement on the digital spectrogram and averaged across all cry expirations. Inter-Cry-Interval (ICI) was the mean duration from offset of one cry expiration to onset of the next; Expiration Duration was the mean duration from onset to offset of all cry expirations. Dysphonation was the mean amount of sonic turbulence in all cry expirations, as determined on a 0–4 point scale used in previous studies [45], averaged across all cry expirations. Table 2 shows the means, standard errors and results of statistical analyses for each of these measures, while Figure 1 shows an example of infant crying with hyperphonation in the latter segments of the crying bout.

**Table 2:** Spectral and Temporal Analysis of Human Infant Cry Sounds.

Measure	Sex	Comparison	Cocaine	Polydrug
Initial F_0_ (Hz)	Male	697.3±86.7	1010.0±115.3	851.9±102.3
	Female	619.5±125.4	672.8±116.3	884.1±96.4
Peak F_0_ (Hz)	Male	728.9±85.5	1204.9±113.7	952.6±100.9
	Female	663.8±123.7	684.8±114.7	926.1±95.1
F_0_ SD (Hz) †	Male	104.8±27.8	235.5±37.0	153.4±32.8
	Female	70.0±40.2	81.0±37.3	125.2±30.9
F_0_ Amp (dB)	Male	59.3±1.6	61.6±2.1	58.3±1.9
	Female	58.1±2.3	51.2±2.2	58.9±1.8
Inter-Cry-Interval (s)	Male	1.4±0.8	1.3±1.1	1.9±1.0
	Female	1.1±1.2	5.9±1.1	2.5±0.9
Cry Expiration Duration (s)	Male	1.3±0.1	1.2±0.1	1.4±0.1
	Female	1.6±0.2	1.4±0.1	1.2±0.1
Number of Cry Expirations †	Male	14.8±1.5	13.5±1.9	12.0±1.7
	Female	12.4±2.1	7.9±2.0	13.7±1.6
Dysphonation	Male	2.2±0.2	2.7±0.2	2.6±0.2
	Female	2.6±0.2	2.1±0.2	2.1±0.2

Note: All values are Mean ± SEM. † denotes analyses conducted on log transformations.

#### Statistical analysis

A 3 (Exposure Group) ×2 (Sex) General Linear Model (GLM) analysis of variance (ANOVA) was conducted on each measure of infant crying using p<.05 for statistical significance. Log_10_ transforms were conducted on measures requiring normalization of their distributions, as denoted in Table 2. Analyses of covariance were conducted using birthweight as the covariate due to its biologically proximal and plausible role in neurobehavioral development and its significant correlation and covariance with the other demographic measures differentiating PCE infants. These analyses showed no reliable effects of birthweight on any cry measures, nor were any differences found in the pattern of results. As such, the presented analyses are based on the unadjusted data. LSD and t-test post-hoc comparisons (with alpha set at 0.05) were used to detect differences between cells in main-effects and interaction-effects, respectively. Chi Square analyses were used for all nonparametric analyses. Analyses were conducted using SPSS statistical software.

### Study 2: Rat pup ultrasonic vocalizations

#### Subjects

Sprague-Dawley rat dams received twice-daily subcutaneous injections of 15 mg/kg cocaine hydrochloride (Sigma, St. Louis, MO) in normal saline (PCE group, n = 34) at approximately 9:00 AM and again at 4:00 PM throughout gestation (GD 1–20) and not thereafter. Saline-treated (SC group, n = 34) dams received twice-daily subcutaneous injections of 0.9% Saline solution (1 ml/kg/injection), and were food-yoked to cocaine-treated dams such that they were only given access to the amount of food consumed by cocaine dams each day. Pups in an untreated control group (UN group, n = 50) received no treatment. At birth, litters were culled to 8 pups per dam, and one male and one female pup were randomly selected for USV recording on postnatal day 5, resulting in a total of 118 pups. Pups were given a series of developmentally appropriate thermal isolation-challenges to induce vocalizations. In a temperature-controlled apparatus, pups were subjected to a 60-min period of habituation at 35.0°C, after which the apparatus temperature was reduced to 28°C (a moderate temperature challenge) for another 60-min. The temperature was then reduced to 21°C (a severe temperature challenge) for a final 60 min. USVs that occurred during two five-minute periods in the final hour were assessed: one after the change to a final temperature was stabilized (minutes 15–20) and one at the end of the hour (minutes 55–60). All animal procedures were approved by the University of North Carolina's Institutional Animal Care and Use Committee (NIH/PHS Animal Welfare Assurance Number: A3410-01) and conducted under federal guidelines for humane treatment of laboratory subjects.

#### Recording and acoustic analyses

Rat pup USVs were recorded and analyzed using previously described methods [44]. Ultrasonic recording equipment included model CM16/CMPA40-5V microphones (Avisoft Bioacoustics; Berlin, Germany) connected to a desktop computer through a National Instruments instrumentation recorder (PCI-6132) with microphones mounted 2cm above the subject platform. National Instruments software (LabView 2009) began acquisition of ultrasonic vocalizations at the session start and terminated at the session end. Digital audio files were down-sampled to 200,000 Hz from 1,000,000 Hz and viewed in a 3-second moving window in a digital spectrogram produced by Adobe Audition.

**Figure 2:**
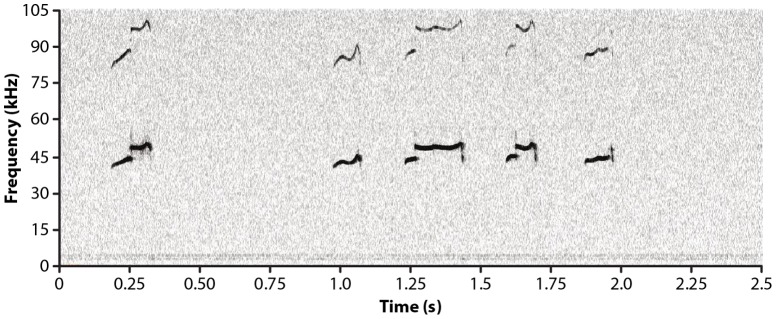
Spectrographic Display of Rat Pup Ultrasonic Vocalizations. This figure shows the qualitative shift in frequency characteristics of shift in rat pup USVs that are similar to the harmonic structure of the hyperphonated cry sounds shown in Figure 4.

A research assistant, trained to criterion and blinded to exposure group and sex of the rat pup, examined all USVs emitted for the presence of a frequency shift. Shift usually occurred in USVs averaging 30 kHz–50 kHz and was defined as an “instantaneous and sudden qualitative shift change(s) in frequency in the absence of a vertical line in the spectrogram, within a single USV”. Figure 2 shows examples of USVs with the frequency shift waveform.

Although not the focus of the present investigation, a secondary set of variables were also compared in analyses to explore their sensitivity to PCE. All USVs were categorized as belonging to the nine categories described by Brudzynski et al. [38] and then consolidated for statistical analyses into five categories based on the complexity of the frequency modulation (change in the direction of the frequency): (1) Low Complexity (categories 0–3); (2) Moderate Complexity (categories 4 and 5); (3) High Complexity (categories 6 and 7); (4) Compound Complexity (category 8); (5) Disorganized (category 9). Further, all USVs in these two five-minute episodes were subjected to spectrum analysis to obtain standard measures of the minimum and maximum F_0_, maximum amplitude, duration, number of harmonics and inter-USV-interval used in previous studies [37]. Measures were obtained using Avisoft-SASLab Pro computer software that automatically detected and logged the selected acoustic features.

#### Statistical analysis

The percentage of rat pup USVs with frequency shift was determined from the audio recordings. The recordings also produced measures of the mean Number of USVs, Duration (s), Inter-USV Interval (s), Maximum Frequency (kHz), Minimum Frequency (kHz), Maximum Amplitude (dBFS), and Number of Harmonics, as well as the percentage of each of the five waveform classifications. All measures were compared with 3 (Exposure Group) ×2 (Sex) Analyses of Variance (ANOVA) followed by LSD post-hoc tests. Alpha levels were set at p<0.05 for all analyses. One pair of male-female rat pups in the SC group was excluded from analysis due to both pups showing unusually significant outlying data (extreme number of vocalizations).

## Results

### Study 1: Human infant crying

**Figure 3:**
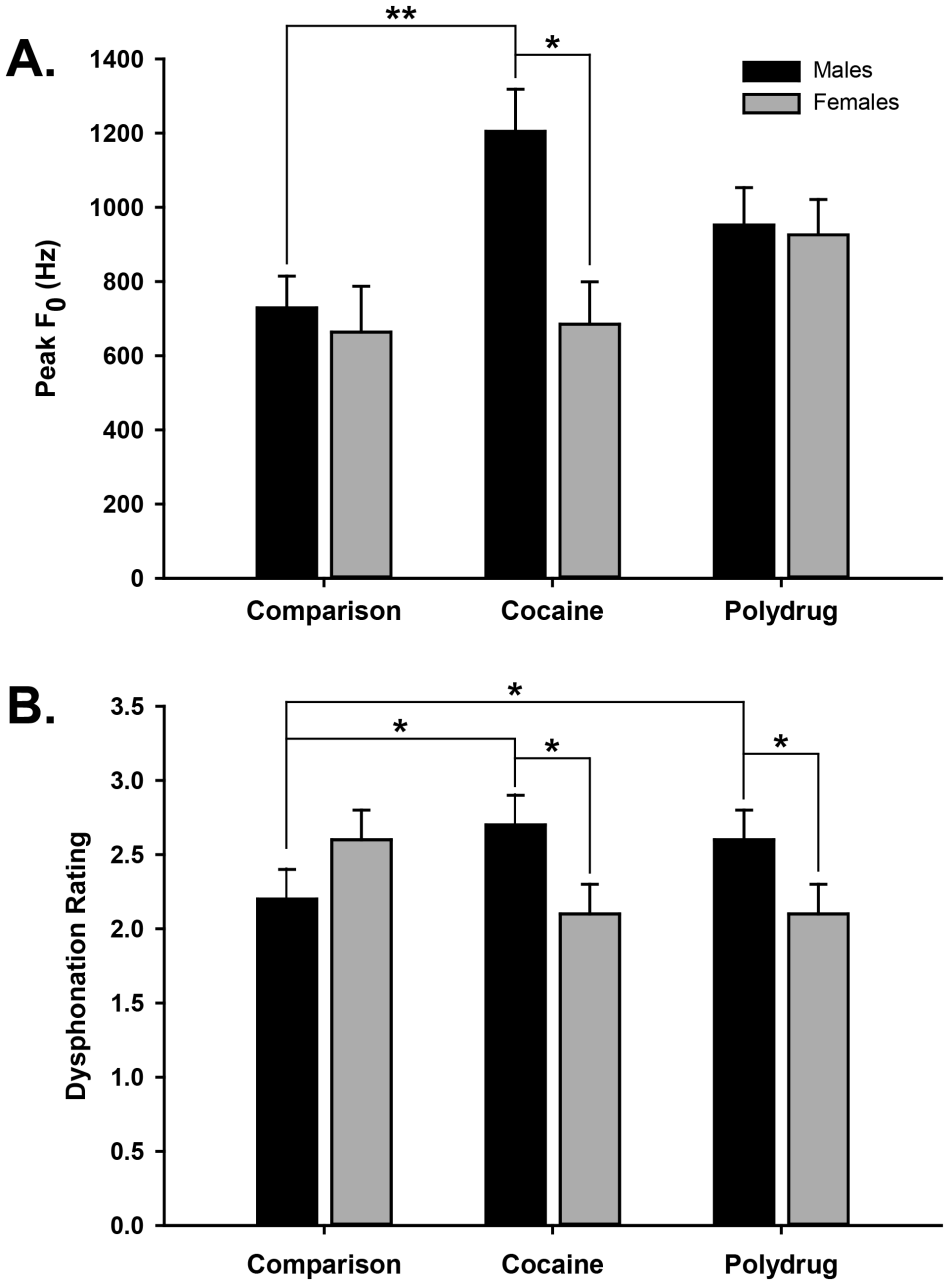
Means, standard errors and significant differences of (A) Peak F0 and (B) Dysphonation for male and female infants in the three exposure groups [Standard Comparison (Control), PCE (Cocaine) and PPE (Polydrug) groups]. The dysphonation score represents the mean rating on a 4-pt scale of the amount of dysphonation in each cry expiration (see text). These acoustic features comprised a cluster of cry measures that differentiated PCE males.

Figure 3 shows sex-dependent effects of cocaine in the analyses of Peak F_0_ and Dysphonation. For the analysis of Peak F_0_, a significant Exposure Group × Sex interaction [F(2, 99) = 3.18, p<.046] and post-hoc analyses showed that PCE males had a higher Peak F_0_ than PCE females (p<.05). No differences were found between males and females in the PPE and SC groups. Additionally, PCE males had a higher Peak F_0_ than SC males (p<.05). No reliable differences were found in Peak F_0_ between males in the PCE and PPE groups, or among females across the three groups. A similar significant Exposure Group × Sex interaction was found for Dysphonation [F(2,99) = 3.66, p<.029]. Post-hoc analyses showed that both PCE and PPE males had cries with more Dysphonation than SC males and more Dysphonation than females in their respective PCE and PPE groups (p<.05) with no significant differences among females. These results indicate that males in both drug exposed groups (PCE and PPE) had similar high-pitched cries with more dysphonation than males in the standard control group, but only PCE males had a higher-pitched cry than PCE females, as well as a higher-pitched cry than SC males.

**Figure 4:**
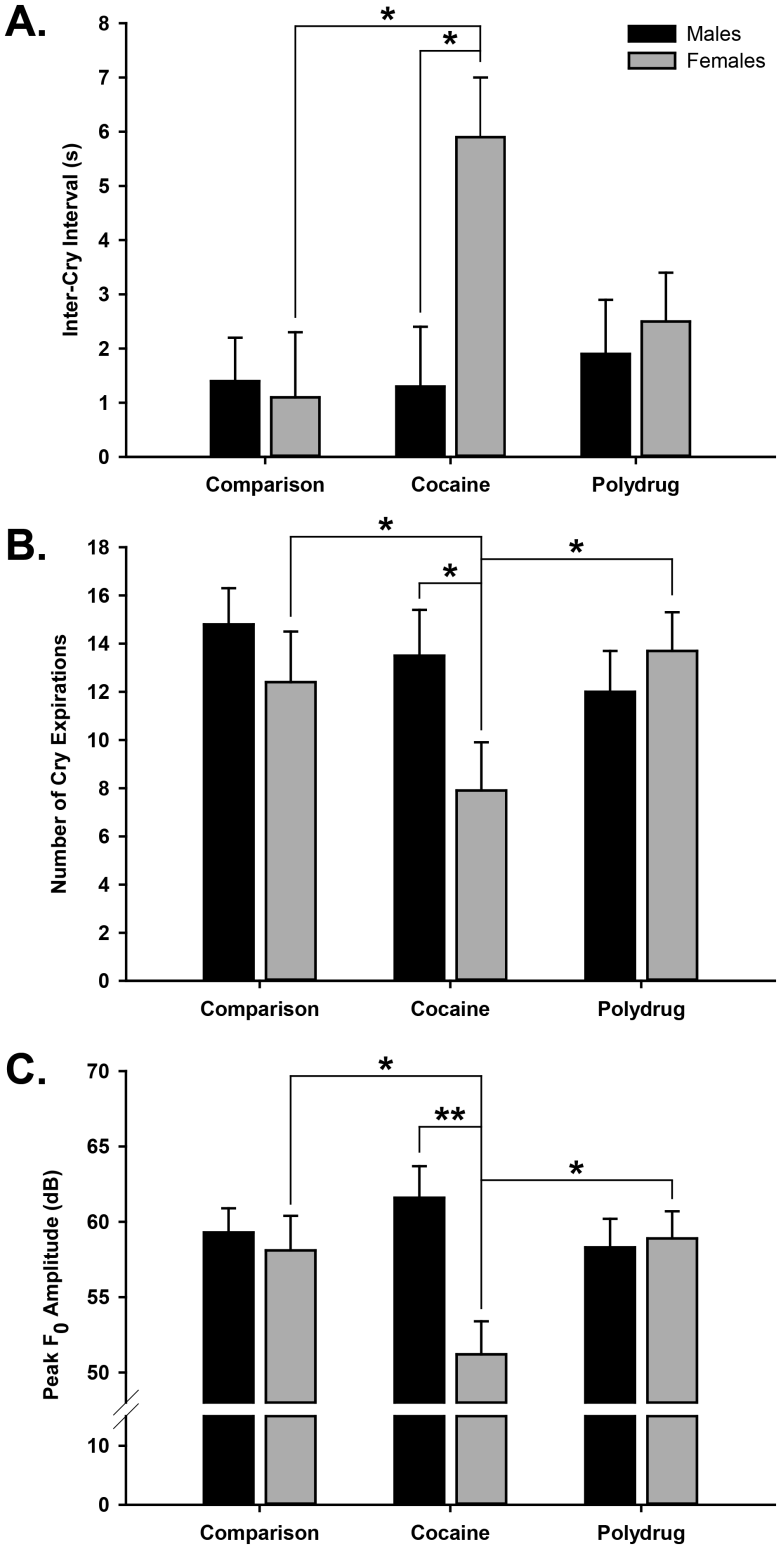
Means, standard errors and significant differences of (A) Inter-Cry-Interval, (B) Number and (C) Peak Amplitude of cry expiratory sounds for male and female infants in the three exposure groups [Standard Comparison (Control), PCE (Cocaine) and PPE (Polydrug) groups]. These acoustic features comprised a cluster of cry measures that differentiated PCE females.

Figure 4 shows sex-dependent effects of cocaine in the analyses of Inter-Cry-Interval, Number of Expiratory Sounds and Peak Amplitude. The Exposure Group × Sex interaction for ICI [F(2,99) = 3.08, p<.05] and post-hoc analyses found that PCE females had longer ICIs than PCE males and females in both the SC and PPE groups (p<.05 all comparisons). The Number of Expiratory Sounds also showed a significant Exposure Group × Sex interaction [F(2,99) = 3.44, p<.036], with PCE females showing a lower Number of Expiratory Sounds than PCE males and females in both the SC and PPE groups (all p's<.05). Lastly, Peak Amplitude likewise showed an Exposure Group × Sex interaction [F(2,99) = 4.24, p<.017], with PCE females having a lower Peak Amplitude of the cry than PCE males and females in both the PPE and SC groups (all p's<.05). No significant effects were found on any of these cry measures for males (all *p*'s>.25) or for the mean Duration of Expiratory Sounds (all *p*'s>.16). Thus, PCE females had longer pauses between fewer cry expirations, which were lower in amplitude, than infants in all other groups.

**Figure 5:**
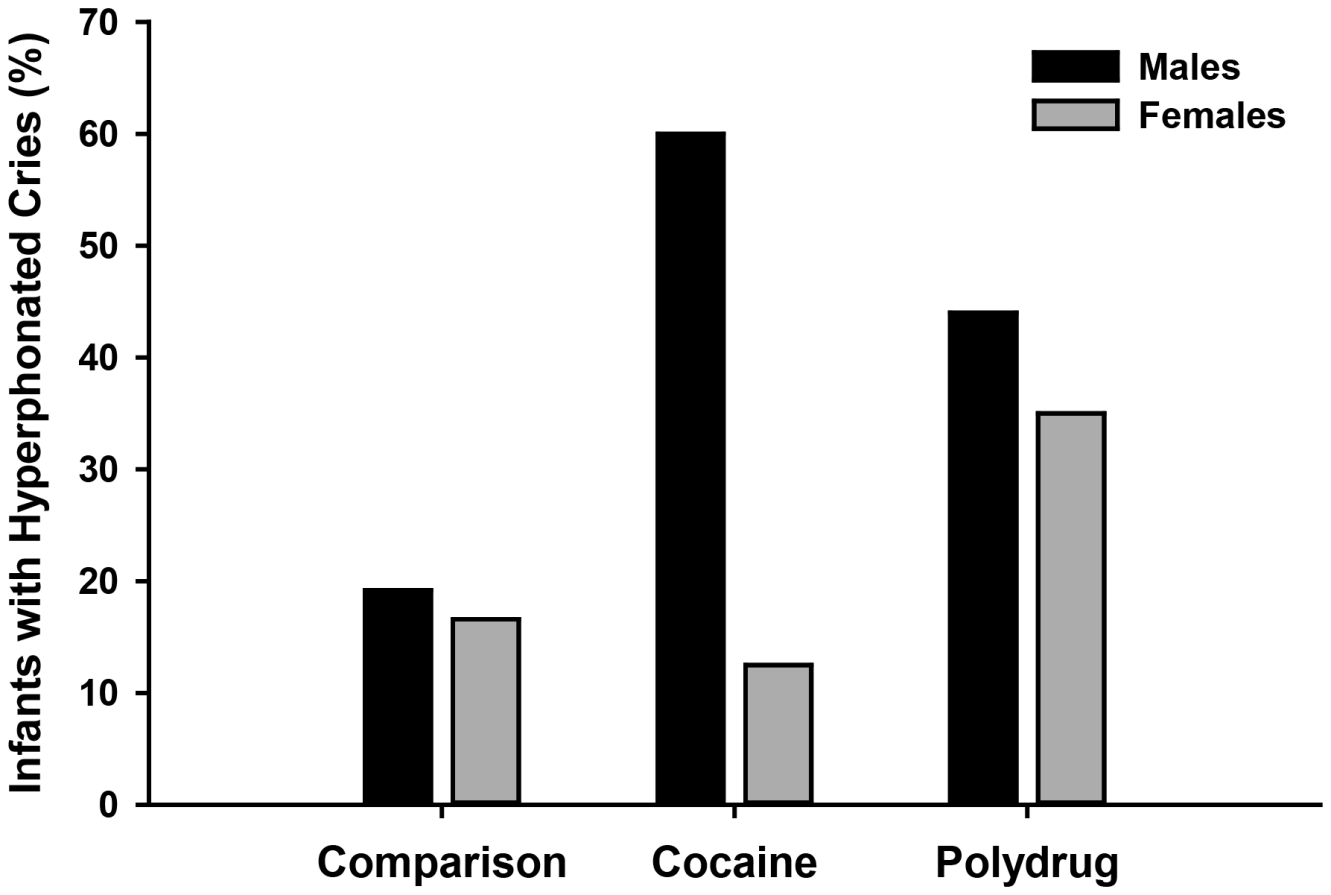
Percentage of infants with hyperphonated cries. This figure shows the increased presence of hyperphonation in PCE and PPE infants. Whereas a similar percentage of PPE male and female infants had hyperphonated cry sounds, PCE male infants showed a higher incidence of hyperphonated cries than PCE females. See text for statistical results.

The multiple measurements of Peak F_0_ allowed us to examine the dynamic nature of the cry along with the distribution of hyperphonation throughout the full cry sound. First, a significant main-effect of Sex for F_0_SD [F(1,99)  = 6.18, p<.015] showed that males had a greater amount of variability in Peak F_0_ over the duration of the cry sound than females. Second, 30.8% (*n* = 33) of all infants had at least one cry expiration with hyperphonation. This acoustic characteristic occurred more often in the cries of PCE (*n* = 11, 35.5%) and PPE (*n* = 15, 39.5%) infants than in the cries of SC infants (*n* = 7, 18.4%) [*X^2^*(2)  = 4.26, p<.049] (see Figure 5). Additionally, males accounted for the majority of infants with cries containing hyperphonation: 9 of the 11 (82%) PCE infants, 8 of the 15 (53.3%) PPE infants and 5 of the 7 (71.4%) SC infants [*X^2^*(2)  = 8.55, p<.01]. Third, hyperphonation occurred in expiratory sounds throughout the 30 s crying bout: (a) in only the initial three cry expirations for 12.1% of infants, (b) in only expirations after the initial three cry expirations for 8.4% of infants, and (c) in both the initial three and subsequent cry expirations for 10.2% of crying infants. The cry expiration with the highest Peak F_0_, independent of whether the acoustic structure was hyperphonated or not, occurred in the portion of crying that followed the initial three expirations for 28.7% of infants, equally distributed among the three exposure groups (PCE = 28%; PPE = 30%; SC = 27.5%). Finding Peak F_0_ in segments other than the initial three cry expirations may have contributed to finding no reliable differences among groups in the Initial Peak F_0_, [F(2,99) = 2.23, p>.113]. These data indicate that cry expirations throughout the crying bout contain important acoustic information that differentiates human infants with PCE.

### Study 2: Rat pup ultrasonic vocalizations

**Figure 6:**
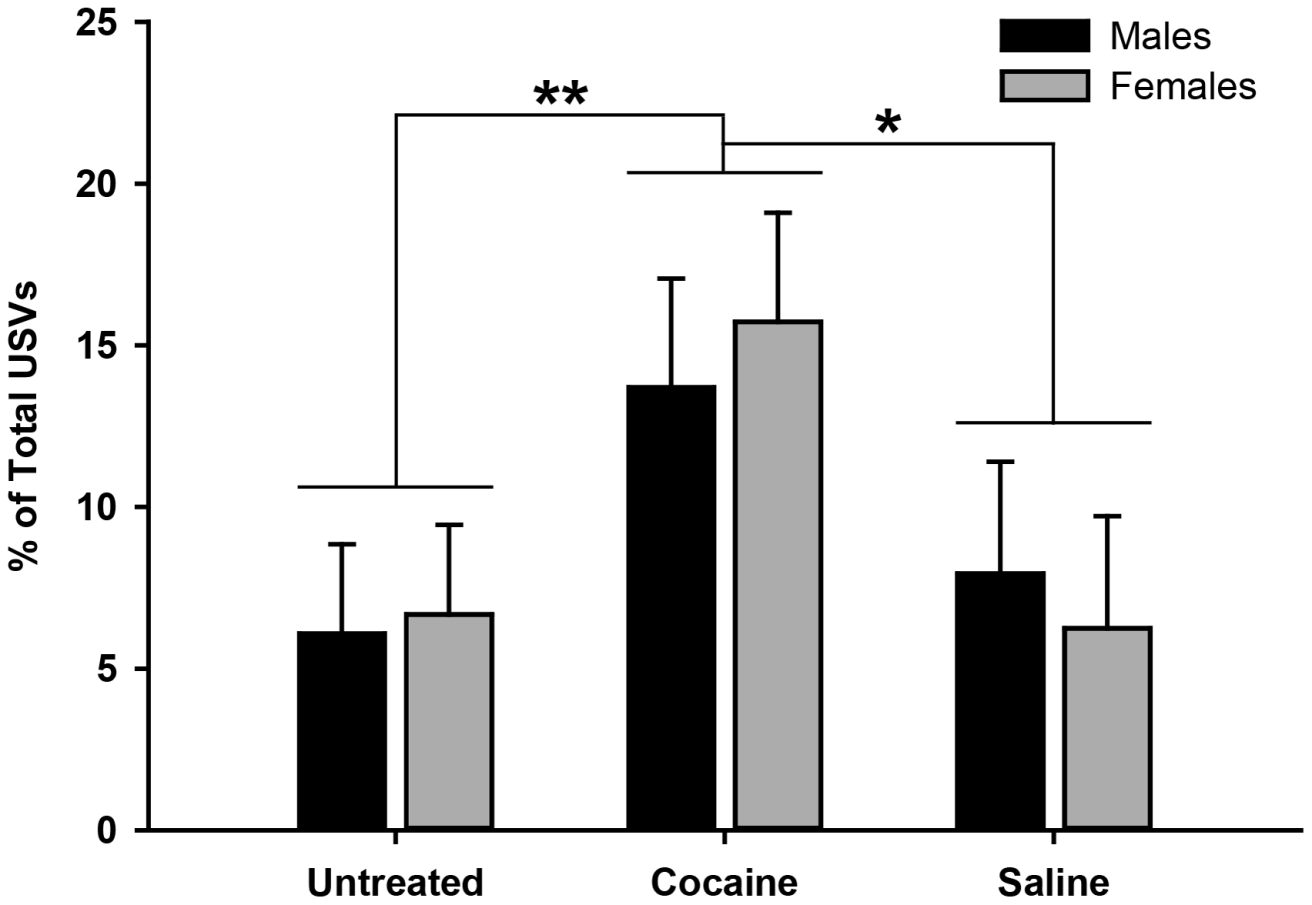
Means, standard errors and significant differences of the percentage of USVs containing Shift for male and female rat pups in the three exposure groups [Untreated (Control), Cocaine and Saline (Control)]. Cocaine-exposed pups had significantly more frequency shifts in their USVs than pups in the Untreated and Saline control groups. No sex differences were found.

The thermal-isolation challenge elicited USVs from pups in all groups (PCE: 23 of 34 (67%); UN: 28 of 50 (56%); SC: 18 of 34 (52.9%). Of those who vocalized, PCE pups averaged 46.6 USVs (range: 1–158); UN pups averaged 55.6 USVs (range: 1–410) and SC pups averaged 66.3 USVs (range: 1–360) during the 10-mins selected for study. The percentage of USVs for each pup that contained shift showed a significant main-effect for Exposure Group [F(2,112)  = 3.72, p<.027], as shown in Figure 6. LSD post-hoc comparisons showed that PCE pups had a significantly higher percentage of USVs containing shift in their acoustic structure than pups in the UN group (*p*<.009) and marginally more than pups in the SC group (*p*<.06). No differences were found between pups in the two control groups in the amount of shift (*p*>.25) nor were there any differences found in the actual number of USVs produced by any of the groups (*p*‘s>.25).

ANOVAs of the secondary set of variables showed no reliable effects for sex, cocaine or cocaine by sex interactions in the percentage of other waveform categories (all *p*'s>.25, see Table 3), nor for any of the spectral and temporal characteristics of the USVs (all *p*'s>.19, see Table 4).

**Table 3:** Percentages of USVs Varying in Waveform Complexity.

Complexity	Sex	Untreated	Cocaine	Saline
Low	Male	17.6±6.1	10.4±6.7	16.5±6.1
	Female	21.0±5.3	20.4±5.9	13.0±10.6
Moderate	Male	30.8±6.9	28.2±7.6	34.9±6.9
	Female	35.3±6.0	24.6±6.6	23.2±12.0
High	Male	31.3±6.5	31.7±7.1	26.0±6.5
	Female	27.4±5.7	28.1±6.3	26.2±11.3
Compound	Male	3.4±2.4	2.6±2.6	8.9±2.4
	Female	3.4±2.1	2.1±2.3	1.5±4.2
Disorganized	Male	1.33±0.95	0.00±1.04	1.08±0.95
	Female	1.06±0.82	1.42±0.91	4.75±1.64

Note: All values are Mean ± SEM.

**Table 4:** Spectral Measures of Rat Pup USVs.

Measure	Sex	Untreated	Cocaine	Saline
Number of USVs	Male	72.5±19.9	47.2±20.8	25.7±19.0
	Female	36.9±17.0	38.1±19.0	35.8±32.9
Duration (s)	Male	0.066±0.010	0.080±0.011	0.069±0.010
	Female	0.072±0.009	0.070±0.010	0.060±0.017
Inter-USV Interval (s)	Male	2.69±6.03	15.45±6.03	18.61±5.75
	Female	9.22±5.29	10.31±5.75	6.33±9.54
Maximum Frequency (kHz)	Male	51.32±3.1	55.47±3.3	49.86±3.0
	Female	57.73±2.7	52.26±3.0	52.41±5.1
Minimum Frequency (kHz)	Male	49.25±2.9	54.16±2.6	46.97±5.0
	Female	51.21±3.2	47.45±3.0	46.72±2.9
Maximum Amplitude (dBFS)	Male	58.8±1.9	59.1±2.0	53.1±1.8
	Female	55.2±1.7	54.8±1.8	56.8±3.2
Number of Harmonics	Male	1.11±0.12	1.10±0.13	1.08±0.12
	Female	0.86±0.11	1.12±0.12	1.22±0.21

Note: All values are Mean ± SEM.

## Discussion

Across species, crying can be conceptualized as a biological siren that reflects changes in arousal and neurobehavioral integrity and then communicates those changes to the social environment [46], [35]. As a biological signal reflecting infant neurobehavioral integrity, we found PCE to be associated with two sex-dependent clusters of measures of human infant crying. The first cluster of cry measures differentiated PCE males and was comprised of two acoustic features derived from the harmonic structure of the power spectrum: Peak F_0_, Hyperphonation, and Dysphonation. The second cluster of cry measures differentiated PCE females and was comprised of three characteristics of the production of crying: the Number and Amplitude of cry expirations and the Inter-Cry-Interval between them. As would be expected from previous studies of licit and illicit prenatal drug exposure [27]–[30], we also found measures of the harmonic structure of infant crying to be sensitive to effects of PPE in the absence of cocaine. A disproportionately higher number of PPE infants (both males and females) had cries with a hyperphonated acoustic structure and PPE males had cries with greater amounts of dysphonation than SC males. Further, experimental evidence of the effects of PCE were found on a measure of rat pup USVs that has a similar acoustic structure to that of hyperphonation in human infant cry sounds. These studies provide the first known direct translational examination of similar acoustic structures of vocalizations of two species in the detection of effects of a similar adverse prenatal condition.

Findings of the present study contribute to a growing literature documenting sex differences in behavioral and physiological reactivity following prenatal substance exposure, in general [47], and prenatal cocaine exposure, in particular [48]–[50]. PCE males are typically described as showing high reactivity to stress-eliciting conditions, such as a higher number of cry cycles in response to heel-lance procedures [48] and greater cortisol reactivity in a paradigm designed to induce stress at 7 months of age [49]. PCE males in the present study showed a cluster of cry features indicative of heightened or excitatory reactivity – cries with a higher pitch and more dysphonation. Importantly, PCE females also showed a distinct and different cluster of cry features depicting a diminished or inhibitory pattern of reactivity - cry expirations that were fewer in number, lower in amplitude and slower in repetition rate. Thus, PCE had perhaps opposite effects on reactivity patterns on male and female infants, as evidenced in the analysis of their cry sounds. Similar excitatory and inhibitory patterns of reactivity in PCE males and females, respectively, have also been found at 13 months of age in a measure of respiratory-sinus arrhythmia [50].

Interestingly, the two clusters of cry measures that differentiated male and female infants in the present study have also been associated with two orthogonal dimensions of neurobehavioral activity in newborn infants [31]. Whereas measures of the fundamental frequency loaded on a neurobehavioral dimension signaling a poorer capacity of the infant to orient to its social environment, measures of cry production loaded on a dimension indicating a poorer ability to regulate behavioral state. As such, the present study provides evidence of sex-specific effects of PCE on physiological reactivity that may be associated with different dimensions of behavior and indicates that these effects are detectable by measures of infant crying at one month of age.

In a direct benefit of the translational analysis, we found independent effects of PCE on an acoustic feature of rat pup USVs that is similar to the acoustic structure of hyperphonation found in human infant cry sounds. Consistent with suggestions that shift may reflect effects of drug exposure on neurobehavioral regulation [43], we found PCE pups to have nearly twice the number of USVs containing that waveform than the two control groups. Also consistent with previous work suggesting that steps or shifts occur more often in a physically intense thermal challenge [42], we found pups in both the UN and SC control groups had 5% to 10% of their USVs with a frequency shift in the acoustic structure as well. To the extent that PCE increases pup sensitivity to environment stressors [51], the increased presence of shift found in USVs of PCE pups may reflect an increased stress response to the physically intense thermal challenge. Whereas sex-dependent effects of PCE were found in the analysis of human infant cries, no such effects were found in rat pup USVs. However, recent work has found sex- and age-dependent effects of PCE in the number, amplitude, harmonics and duration of USVs of older rats [52]. Lastly, no significant effects of PCE or sex were found for any of the other USV waveform categories or the standard spectral and temporal measures analyzed in the present study. Thus, the presence of shift in rat pup USVs may uniquely provide a sensitive translational measure of neurobehavioral integrity following prenatal cocaine exposure.

Our understanding of the physiological mechanisms underlying effects of PCE on vocalizations varies between the two species examined in this study. For human infants, pathophysiological pathways by which PCE affects neurobehavioral function [11] and the physioacoustic basis for variations in human infant cry sounds [53], [54] have been well-described. Variations in F_0_, for example, have been suggested to result from activity of lower brainstem mechanisms that control tension of laryngeal muscles through the vagal complex and phrenic and thoracic nerves. Instability in these neural mechanisms is thought to produce hyperphonation, while dysphonation, amplitude and temporal morphology cry reflect inhibitory and excitatory aspects of respiratory function. In other species, the specific effects of PCE on nervous system development, including the mesolimbic dopamine system [55], have been more fully described, but less is known about how PCE specifically affects production of rodent USVs in general, and waveform shifts in particular. While the vocal anatomy of the rat pup is similar to other mammals who have vibrating vocal folds at the top of the trachea, a current consensus is that ultrasonic tones result from a still unknown whistle-type mechanism [40], [56]. Some suggest effects of PCE on USVs may result from stimulation of endogenous opioid systems [57], activation of “pleasure” systems [43], and effects of PCE on maturation of the central medial hypothalamus and central amygdala [53]. Future work that examines the bases of shift and other variations in acoustic features of USVs will contribute to our understanding of the translational value of these analyses.

As a biological siren, variations in the sound of crying are also salient social signals [46] that may contribute to problem mother-infant interactions that are often a part of the context that guides developmental outcome in cocaine-exposed infants [19]–[22]. For example, the number of expiratory cry sounds, slower cry repetition rate [58] and variability in Peak F_0_ [59] found in the present study have been shown to create specific perceptual responses in potential caregivers. Hyperphonation, in particular, is a powerfully salient vocal signal. In many mammals, vocalizations that induce attention and arousal often have sharp onsets, dramatic fluctuations in frequency and amplitude and either shorter or longer, upward sweeps in frequency [60]. These vocalization patterns are characteristic of both hyperphonation and shift. The attention and arousal elicited by hyperphonated cry sounds are mediated by two orthogonal perceptual dimensions that may provide the basis for ameliorative care from some caregivers and heightened distress and withdrawal from others [46], [61]. Typical of the latter response set, cocaine-using mothers report they would give less nurturing responses than comparison mothers to infant cries, especially as the sound of crying increases in fundamental frequency [62]. In this case, hyperphonated cries may contribute to maternal withdrawal and other behaviors that put the infant with PCE at further risk for detrimental social interactions, including physical child abuse and neglect [63], [64]. Similarly, variations in acoustic and temporal features of rat pup USVs are part of the sensory environment that elicits differential caregiving responses from the dam [65], yet no known work has yet examined the social significance of the shift waveform examined in this study. Future work would benefit from the exploration of the functional significance of this unusual acoustic characteristic.

Several limitations of the methods should be considered when interpreting the results of these studies. The Time Line Follow Back and urine analyses do not provide a full understanding of the timing and amount of human maternal cocaine-use during pregnancy. The two species differed in the vehicle by which cocaine-exposure was delivered, the methods by which vocalizations were elicited and the developmental age at which vocalizations were recorded. Although there was no evidence of such, variations in spectral and temporal characteristics of the cries of infants with PCE could also be possibly due to unknown adverse conditions that occurred during the first postnatal month. The effect of the long observation period on the rate of rat pup vocalizations in this study is unknown. Last, exploratory analyses of the secondary set of measures of rat pup USVs were conducted to “rule out” their sensitivity to the effects of PCE. These analyses increased the number of statistical comparisons and possibility of a Type I error in which significant findings may have occurred by chance. While none of these secondary, exploratory analyses were statistically significant, most of the primary measures that were the focus of our study were statistically reliable.

In conclusion, these two studies provide the first known direct translational examination of similar acoustic structures of vocalizations of two species in the detection of effects of a similar adverse prenatal condition. A novel set of measures in an analysis of an extended period of human infant crying uncovered different effects of PCE on patterns of reactivity in male and female infants. The analysis of shift in rat pup USVs distinguished PCE pups from controls – in the absence of variables that often confound results in studies of human infants. While the context and mechanisms producing vocalizations differ between human infants and rat pups, there is an intriguing similarity of acoustic structures following prenatal cocaine-exposure that differentiated their vocalizations. Further, while many challenges preclude making direct homologous comparisons between the distress vocalizations of human infants and rat pups, a recent review concludes that there is ample evidence for employing apparent similarities in research on the biological effects of PCE on animals and humans, as well as contributing effects of the rearing environment [55]. Within this context, the variations in the sounds of infant crying and rat pup USVs may also have important functional value, impacting the rearing environment. As such, the analysis of cry vocalizations may have significant translational value by affording a window into both the neurobehavioral and social complexities underlying the development of infants with prenatal cocaine and other drug exposures. The methods and findings presented here may provide the basis for future research questions and a foundation for future translational analyses into a possibly wide range of developmental disorders.

## Supporting Information

File S1 **Raw data from the experiment.**
(ZIP)
